# A Video Interview With Caroline A. Glicksman: My Second Thoughts About Intraoperative Implant Sizing

**DOI:** 10.1093/asjof/ojac094

**Published:** 2022-12-29

**Authors:** Caroline A Glicksman, Lorne King Rosenfield

**Affiliations:** Hackensack Meridian School of Medicine, Nutley, NJ, USA; Department of Plastic Surgery, University of California San Francisco, San Francisco, CA, USA

For this edition of *Second Thoughts on First Thoughts*, we are treated to the wisdom of a true master of breast surgery, Dr. Caroline A. Glicksman (Video). She recounts her second thoughts on an age-old strategy of breast implants: intraoperative sizing. So, take a break for a few minutes to learn what Dr. Glicksman learned about this topic and how her practices have changed forevermore. The interview follows the format of the 5 Ws in investigative reporting: Who, What, Where, When, and Why? We hope you enjoy this brief but edifying interview.

## Supplementary Material

ojac094_Supplementary_Data

